# Hydrogen sulfide: both feet on the gas and none on the brake?

**DOI:** 10.3389/fphys.2013.00002

**Published:** 2013-01-25

**Authors:** Kenneth R. Olson

**Affiliations:** Department of Physiology, Indiana University School of Medicine - South BendSouth Bend, IN, USA

**Figure 1:**
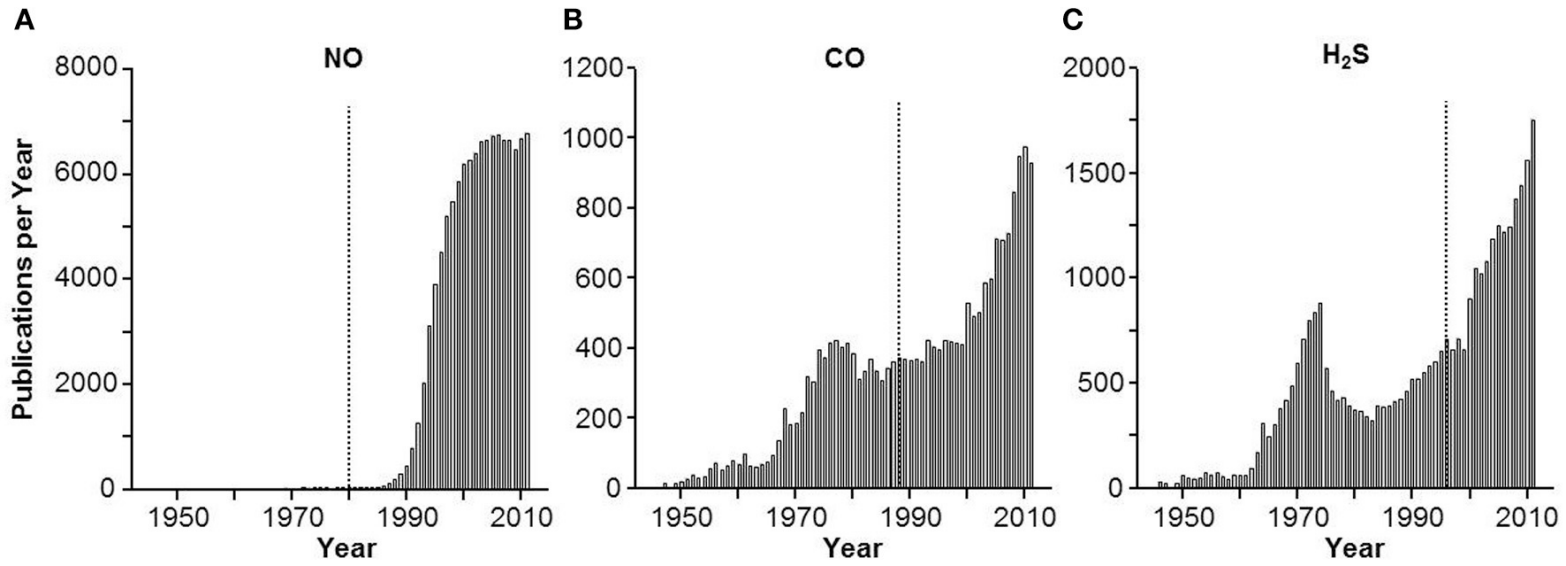
**Frequency histogram of the number of yearly publications on (A) nitric oxide (NO), (B) carbon monoxide (CO) and (C) hydrogen sulfide H_2_S**. Vertical lines indicate approximate year of the first description of a biological effect of the gas. Approximately 10 years passed before NO and CO caught on as biologically relevant signal molecules, acceptance of H_2_S was much quicker.

I remember as a neophyte academician in the early 80's the excitement and at times heated discussion generated by nitric oxide (NO). How could this toxic and relatively unheard-of gas possibly be a regulatory molecule? Even its discovery was the result of a laboratory mistake (Furchgott, 1995). As with any revolutionary idea, there were both zealous aficionados, naysayers and folks in between that “just wanted more proof.” As a result, it took nearly 10 years for this field to reach its log growth phase (Figure 1A); the fact that the number of yearly publications has now leveled off at approximately 7000 per year is probably more attributable to resource limitations than to enthusiasm. The physiological relevance of carbon monoxide (CO) was realized shortly after (Wu and Wang, 2005) but again there was a similar lag between discovery and acceptability as the scientific community tried to separate toxicology and pharmacology from physiology (Figure 1B).

Hydrogen sulfide (H_2_S) was the third gas to be elevated to signaling status, i.e., a “gasotransmitter” (Wang, 2002). H_2_S has the same toxic pedigree as NO and CO (Guidotti, 2010) but it has not received the degree of initial skepticism that accompanied the latter two. As shown in Figure 1C, following the initial demonstrations of H_2_S signaling in the nervous and cardiovascular systems by Kimura's group (Abe and Kimura, 1996; Hosoki et al., 1997) the lag-time for H_2_S to catch on in the scientific community took about as long as the time for these two seminal papers to circulate. Within 3 years the log phase of H_2_S publications had begun, and it continues to this day.

It is not surprising that H_2_S was greeted with enthusiasm; gaseous signaling molecules were no longer novel, the implications of toxicity arising from excess signal were obvious, and a precedent for therapeutic potential (and profit) were firmly established. Add to this the fact that exogenous H_2_S appears to affect virtually every physiological system, it has potentially more biochemical diversity than the other gases, journals are eager for reviews (132 in 2011 and 93 as of the time of this writing in 2012), and away we go. The thought that the Emperor may not be wearing any clothes, and may even have a few warts, seems to be lost in the gala of the parade to the publisher and the patent office.

This is not to belittle the considerable potential of H_2_S research, there have been momentous gains, and undoubtedly more to come. Exogenous H_2_S affects virtually every organ system and physiological process to which it has been applied. Many of the effects of H_2_S are supported by compounds that are thought to inhibit or augment endogenous H_2_S production (See reviews; Caliendo et al., 2010; Kimura, 2010; Szabo, 2010; Olson, 2012b; Wang, 2012). The most notable among these are the cardiovascular system, where H_2_S mediates systemic vasodilation and pulmonary vasoconstriction, angiogenesis, oxygen sensing and is cardioprotective, the nervous system where H_2_S affects neuronal signaling, contributes to involved retinal function and may be involved in degenerative diseases, and the gastrointestinal (GI) tract where it contributes to signaling, insulin release, and metabolism. H_2_S appears to be anti-inflammatory in most tissues, although it may be inflammatory in some. Contrary to common thought, H_2_S itself does not readily react with reactive oxygen species (ROS) but it does stimulate glutathione production and augment reducing equivalents in the central nervous system (Kimura et al., 2010). Even novel signaling mechanisms whereby H_2_S interacts with the reduced (sulfhydration) or oxidized sulfur of cysteine to activate or inactivate proteins have been described (Mustafa et al., 2009; Tao et al., 2012). As well-illustrated in this issue, it is also becoming increasingly evident that the many of the biological effects of H_2_S are mediated directly through H_2_S interactions with ion channels (see also; Tang et al., 2010; Munaron et al., 2012; Peers et al., 2012).

Sulfide salts, such as NaHS and Na_2_S, have been historically used to rapidly generate H_2_S but this occurs at an uncontrolled rate and their purity may be problematic. These salts are gradually being replaced by the next generation of compounds that slowly release H_2_S. The clinical applicability these true H_2_S “releasing” drugs is now becoming evident and they offer additional advantages in that they can be combined with other, unrelated drugs and provide a multi-focal therapeutic approach (Caliendo et al., 2010; Olson, 2011; Kashfi and Olson, 2012). Already, H_2_S-releasing drugs have been combined with a variety of compounds including virtually all non-steroidal anti-inflammatory drugs (NSAIDs), sildenafil, levodopa, the anti-glaucoma drug lantanoprost, and the angiotensin-1 receptor antagonist losartan to name a few. The ability of some of these H_2_S-releasing drugs to uniquely counter the adverse effects of aspirin and other NSAIDs on the GI tract or to treat other inflammatory process such as inflammatory bowel disease (IBD), irritable bowel syndrome (IBS) and acute and chronic joint pain are nearing or already in initial clinical trials. A third generation of compounds that can be combined with drugs such as NSAIDs and release both H_2_S and NO, the so-called “NOSH” compounds may further increase potency and versatility; preliminary studies suggest they may be especially effective in the treatment of cancer (Kashfi and Olson, 2012).

A better understanding of H_2_S biochemistry and metabolism has also lead to promising therapy (Viscomi et al., 2010; Drousiotou et al., 2011). Ethylmalonic encephalopathy (EE) is an autosomal recessive deletion in the ETHE1 gene that encodes a mitochondrial sulfur dioxygenase. EE is typically fatal in the first decade of life. Patients with EE present with greatly elevated urinary and tissue thiosulfate concentrations and increased tissue H_2_S, presumably due to the inability of cells to efficiently metabolize H_2_S. This condition has recently been treated by oral administration of the bactericide metronidazole, to inhibit H_2_S production by colonic bacteria, and *N*-acetylcysteine. The latter acts as a membrane-permeable precursor of reduced glutathione (GSH) and appears to combine with H_2_S to form a non-toxic persulfide, GSSH. Targeting another mitochondrial enzyme, sulfur quinone oxidoreductase (SQR), which catalyzes the first step in H_2_S oxidation, may be a future therapeutic option (Linden et al., 2010).

The intent of this opinion is not to be a nayser, but just to request more proof that while we are going full-speed ahead we are also going in the right direction and not out of control. Clearly, we don't need proof that exogenous H_2_S activates/inhibits numerous biological systems, or that these systems can be modulated by fiddling with H_2_S metabolism, or that H_2_S has specific molecular targets, there are at least 225 reviews to substantiate these. However, we do need to critically examine a number of practical and technical issues. These include the promiscuity of many “selective” inhibitors of H_2_S biosynthesis, separating physiological from pharmacological effects of H_2_S, dosing issues, and measuring H_2_S in tissues and blood. Obviously, all are related; I will focus on the latter two.

H_2_S is a gas and we need some justification for ignoring its ephemeral nature; the half-life of H_2_S in open containers is a few scant minutes, even less when aerated (DeLeon et al., 2011). When we expose cells and tissues to H_2_S for hours and days in tissue culture or organ baths what are we looking at? Is this why we need to expose tissues to excessive H_2_S concentrations that can't possibly exist naturally? And where do we separate physiology from toxicology?

More importantly, however, we really need proof that what we are measuring in tissues and blood is actually H_2_S. This has profound implications in perhaps the most exciting avenue of H_2_S research, its therapeutic potential. My biggest concern is that if we do not do this right it can have disastrous consequences resulting in missed diagnoses and inappropriate therapies. I will give three examples, one where a simple calculation can be used to tell us if tissue H_2_S values are realistic, a second that will (hopefully) red-flag plasma and blood H_2_S measurements and a third that serves as an excellent example of how critical thinking can put the brake on a potentially dangerous clinical application.

First, lets look at tissue H_2_S concentrations. Most studies report tissue H_2_S concentrations anywhere from 1 nmol/mg protein to up to values well in excess of 10 μmol/mg protein. As I have previously calculated (Olson, 2012a) if one assumes a cell is 15% protein and 70% water then 1 μmol H_2_S/mg protein would result in a cytosolic concentration of 214 mmol/l! Even 1 nmol/mg protein in a cell is still be more than five times the toxic level. How can these values be so high? There are at least two reasons. First, most methods require incubating the tissue for 30 min or more under anoxic conditions. These conditions prevent normal H_2_S oxidation while H_2_S production continues unabated. Second, the most commonly used methods, methylene blue, monobromobimane and ion-selective electrodes (ISE) measure more than just H_2_S; and it's generally not clear what many of these other molecules are (Olson, 2012a). The alkaline antioxident buffer used with the ISE generates H_2_S from cysteine in serum albumin (Whitfield et al., 2008).

Even more troubling are H_2_S measurements in plasma and blood. H_2_S is commonly measured in plasma using the above methods or in headspace gas (typically after equilibration for 30 min or more under anoxic conditions) and concentrations from 1–30 μmol/l are common, although some go as high as 300 μmol/l. These are unrealistic because of the reasons described above, and as so elegantly shown by Furne et al. (2008), the human nose can detect 1 μmol/l; blood obviously doesn't smell like rotten eggs. While these erroneous values are a detraction in laboratory experiments, they are potentially dangerous when used diagnostically. And the latter is becoming more common. I offer a simple challenge to any laboratory that is interested in, or currently measuring H_2_S in blood; in addition to making your standard curve in buffer, make another standard curve in whole blood and compare the two. I guarantee the values obtained in the plasma will not correlate with buffer values and, in fact, there will be no measurable H_2_S in the former because red blood cells avidly remove H_2_S (Whitfield et al., 2008).

There is considerable, and alarming interest in using blood H_2_S measurements for clinical diagnosis and a variety of pathological conditions have been correlated with altered plasma H_2_S. Here I will give a single example. Goslar et al. (2011) used the standard methylene blue method to measure total plasma sulfide in patients admitted to the intensive care unit (ICU) with various forms of shock. Presuming plasma pH is 7.4 this would be ~20% H_2_S gas; in acidosis the percent H_2_S gas will increase. Goslar et al. (2011) reported that plasma sulfide was lower in survivors than non-survivors (13 vs. 32 μ M) and they concluded that plasma sulfide could be used as a predictor of mortality in the ICU. This concept was critically tested in a rat model of hemorrhagic shock by Van de Louw and Haouzi (2012) who found no evidence for such a correlation and, in fact, the methylene blue method measured plasma turbidity, not sulfide.

This Opinion is not meant to discourage research in this exciting field but to encourage critical thinking about how we are generating information and interpreting it. Clearly, these are exciting times. However, it may be necessary now and then to apply the brakes on exuberance to keep things from getting out of control. These points are further detailed in several recent reviews (Olson, 2009, 2011, 2012a; Linden et al., 2010).
